# Fatal Errors

**Published:** 1882-02

**Authors:** Ad. Lippe

**Affiliations:** Philadelphia


### FATAL ERRORS.

AD. LIPPE, M. D., PHILA.

It is a fatal error to attempt to prove the correctness of erroneous propositions by falsified quotations.

The attention of the profession is called to these auxiliary and supplementary means, false quotations, used to make the erroneous propositions, of some professing homœopathists, appear as if they were sustained by the Master. It is especially wrong to do this when an open question like posology is dragged before the reading public again, and when the point to be proved is not a question of moment at present.

In the *Medical Call*, a homœopathic quarterly, a correspondent, it appears, has insinuated to the editor that he knew a better Homœopathy forty years ago, than is promulgated by that journal at present. The editor calls him a forty-year-fossil (122nd p. 4th No. of the first volume of said journal). "In approval of the practice of those careful, conscientious physicians, who prefer generally the low potencies" (who ever denied any one the right and freedom to use them?), the editor refers to Hahnemann's Materia Medica Pura, *edited* (should read *mal-translated*) by Charles Julius Hempel, M. D., and referring to the preface to *Cannabis*, they make Hahnemann say: "For a long while I have used a small portion of a drop of the *undiluted tincture* at a dose." What did Samuel Hahnemann really and truly say? It is this: "For a long time I used the *smallest* part of a drop of the undiluted alcoholic tincture of the juice of *Cannabis sativus;* but the higher and till now the highest dilution and potency, the thirtieth, develops the medicinal virtues of this plant in a much higher degree." The first part of this sentence would have fully shown that the conscientious gentlemen who prefer the lower potencies are sustained by Hahnemann's own practice, but unfortunately for the editor and fortunately for that fossil, Hahnemann declares that the 30th potency of this plant *develops,* note it well, *develops* the medicinal virtues of this plant in a much higher degree. The *Medical Call,* after committing this fatal error, says, we hereby declare "a firm conviction, that the potency is, and ought to be, a matter of individual, personal judgment." We fully agree with the learned editor, *provided,* the individual, personal judgment is the result, not of mere theory, hypothesis, or speculation, but of honest, painstaking experiment; such an experiment as induced Hahnemann to give the world the results of his experiments in the last part of the above quoted sentence.

An equally objectionable *fatal error* is an attempt to cover up new departures by the falsification of history. We have had occasion recently to point out the only possible *motive* which could have induced the compiler of the "Historical Volume of the World's Homœopathic Convention," held at Philadelphia in 1876, published in 1881. This was to put on record (see page 801) as a homœopathic institution, the late "Penn Medical University" (an eclectic school). Inexcusable as this bold attempt was to foist eclectic schools on homœopathic institutions, the entirely incompetent compiler seems utterly ignorant of a history already fully dealt with by the late Dr. Carroll Dunham. In the address before the "World's Homœopathic Convention of 1876," Dr. Carroll Dunham said "*We have seven Colleges, exclusively homœopathic, enjoying equal privileges with any other medical colleges in this country; and two State universities*" *etc.* By what possible legerdemain then can an eighth institution, *not exclusively homœopathic*, be put on record as a homœopathic institution? Does the "Historian" propose to prove that the late Carroll Dunham was in error? or does the "Historian" propose to correct the statement made by Dr. Dunham in his address? Really the times seem to be out of joint.

It is a fatal error to believe that there ever existed, or could exist, or will hereafter exist, specific remedies for specific diseases. There is a hunt for them kept up by all sorts of brainless physicians, by quacks and by professing homœopathists sporting the pathological livery. In the *Medical Advance*, October, 1881, is to be found a very interesting and amusing paper, entitled "How Much of this is Fraud?" by Dr. T. Mills Fowler, Dubuque, Iowa. It appears that an Englishman sold to professing homœopaths a specific for rheumatism with the sole right to use it in the county in which the purchaser resided. The Englishman exhibited a long list of purchasers from among the ranks of homœopathic practitioners and was only found out when he offered his nostrum to two homœopaths in the same town; having sold it to one, he attempted to sell it again and was found out. Precious representatives of the homœopathic school of medicine. Followers of Hahnemann purchasing a specific for rheumatism! ! ! Comment is unnecessary.

